# Social Validation Influences Individuals’ Judgments about Ownership

**DOI:** 10.3389/fnint.2018.00002

**Published:** 2018-01-30

**Authors:** Leandro Casiraghi, Gustavo Faigenbaum, Alejandro Chehtman, Mariano Sigman

**Affiliations:** ^1^Instituto de Física de Buenos Aires, Universidad de Buenos Aires, Buenos Aires, Argentina; ^2^Consejo Nacional de Investigaciones Científicas y Tecnológicas, Buenos Aires, Argentina; ^3^Laboratorio de Neurociencia, Universidad Torcuato Di Tella, Buenos Aires, Argentina; ^4^Facultad de Humanidades, Artes y Ciencias Sociales, Universidad Autónoma de Entre Ríos, Gualeguaychú, Argentina; ^5^Escuela de Derecho, Universidad Torcuato Di Tella, Buenos Aires, Argentina; ^6^Escuela de Negocios, Universidad Torcuato Di Tella, Buenos Aires, Argentina

**Keywords:** property, ownership, conflict resolution, legal thinking, intuitions

## Abstract

In all domains, from informal to formal, there are conflicts about property and ownership which resolution demands consideration of alleged claims from more than one party. In this work we asked adults (*N* = 359) to judge cases in which a character held a property claim over an item, but is challenged by a second character who holds a different, subsequent claim over it. The specific goal of this work is to investigate how the resolution of such conflicts depends on the social endorsement of ownership claims. To achieve this aim, we designed variations of conflictive situations over property in which we manipulated details regarding the knowledge of the second agent of other third-parties about the first agent’s actions. In essence, our questions were: if an agent claims ownership of something which has a previous property claim on (1) does it matter whether said agent knew of the first’s agent actions or not? And (2) does it matter whether third parties were aware or notified of the first one’s claim? The results confirm that adults resolve the settling of property rights based not only on the nature of ownership claims but also on the social acknowledgment of such claims, in accordance with what is stipulated in legal systems worldwide. Participants considered the second character in the stories to hold a lesser right over the object under dispute when she knew of the first character’s claim. Participants also considered that the first character’s claim was reinforced when there were witnesses for her actions, but not when third parties were merely communicated of such actions. This is the first study to our knowledge that studies how social validation of ownership claims drives adults’ judgments on property claims.

## Introduction

The notion of property, and the rules that determine who owns what and why, are cornerstones of every human society. Humans, as well as other animals, typically engage in possessive behaviors (Brosnan, 2011; Rochat, 2014). Moreover, insofar as individuals aim at exclusive control over scarce goods, other individuals are seen as competitors and as potential enemies to be excluded. And yet, humans are also highly social beings who balance their aggressive and competitive tendencies with other, equally powerful, cooperative inclinations (Tomasello, 2009). Indeed, property regimes rest on an underlying thread of cooperative behavior (Rose, 2007). The institution of ownership is only possible because human agents abide by the rules in force and behave in predictable ways. “Property, as an institution, requires stability in people’s expectations about their own and other people’s claims” (Rose, 2007, p. 1906). Understanding how we humans reason and behave in this social-normative domain helps us to understand how we are capable of thriving together in society. In this work, we study the concept of ownership as a societal construct which relies on and demands validation from the social environment. Specifically, we look at how acknowledgment about other people’s property claims affect the way we consider arguments over ownership between third parties.

Usually, determining ownership involves a decision process in which different arguments may be in conflict. Imagine that a person collects some rare kind of wood in the forest and leaves it near a road where someone else finds it and builds a chair with it. Now the identity of the disputed item has changed (from wood to a chair), but the discoverer of the wood still has a valid claim over it. Who should keep the chair, the person who found the wood or the person who built the chair? This concise narrative confronts the principles of discovery and previous possession with the principle of creation (and hence, of first possession of the created item). Subjective arguments (for instance, the knowledge of the intention a person has about something) can also come into play in the judgment of ownership. For example, in the previous story, one relevant issue may be whether the person who built the chair knew that the wood was there because someone else had just gathered it for using it in the future with another purpose.

Such considerations involve a “mental” aspect, such as the possible thoughts and knowledge of the character who creates the chair in the example above – but also a “communicative” aspect. Some legal theorists have in fact outlined the importance of communication in the setting of property. For example, Carol Rose explains that Common Law defines the act of possession as “a kind of statement” and that the possessor’s acts must be a declaration of her intent to appropriate (Rose, 1985). Similarly, in the 1931 *Clipperton Island* arbitration it was stated that there “is no reason to suppose France has subsequently lost her [title over the island] by *derelictio* [i.e. abandonment], since she never had the *animus* of abandoning [it], and the fact that she has not exercised her authority there in a positive manner does not imply the forfeiture of an acquisition already definitively protected” (Emmanuel, 1932, p. 394). Thus, possession is “a kind of speech, with the audience composed of all others who might be interested in claiming the object in question”; possession as the basis of property amounts to “yelling loudly enough to all who may be interested” (Rose, 1985, p. 79, p. 81). Rose’s point is that, for the law, physical possession is not enough and appropriate communication is necessary.

The motivation of this study can be seen as an effort to inquire how these theories of property resonate with folk intuitions, by asking participants to judge conflicts of property in which the communication intention of one agent, or the knowledge of previous possession of another agent are manipulated. Specifically, we ask whether the following possibilities hold in the intuitive reasoning of adult individuals: (a) the consideration of claims, including history of possession, determine the resolution of conflicts; (b) declarative speech *per se* consolidates possession and protects ownership from a second possessor’s claim; or (c) factual confirmation of possession claims (either by the second possessor or by other social agents) is necessary to warrant an original possession claim against subsequent claims.

To achieve the goals of this work, we asked adult volunteers to judge over concise situations in which an individual claims the property of something which ownership has already been claimed by a first agent. Our study required the presentation of conflicts between two people who hold *different* and *sequential* claims over a certain item, over which volunteers could judge. Using *different* claims allowed us to test the extension of the studied effect under different scenarios (also, the presentation of equal claims from both characters could induce volunteers to consider that one of them is lying). They also needed to be *sequential* in time to allow for the inclusion of the factor of knowledge regarding a first claimant.

The cases were based on three specific types of ownership claims: *discovery*, *creation*, and *adverse possession*. These three were selected because they represent claims of property which are ubiquitous in legal systems worldwide. *Discovery* represents an absolute case of first possession by finding something for the first time. *Creation* combines first possession of the object created (which did not exist before) with a possible previous history of possession/ownership of the raw materials used. *Adverse possession*, or *occupation*, as we will refer to it from now on, necessarily implies the settling on a property which was occupied or controlled by a previous owner. Such differences provide a wide range of situations on which to study whether and how the validation of property claims from a first actor either by the second actor or by third-parties affects judgments in particular arguments over property.

## Materials and Methods

### Participants

Participants in the study (*N* = 359) were recruited through our laboratory’s social networks web pages. Volunteers participated through anonymous online forms after completing an informed consent about the objectives of the investigation in accordance with the Declaration of Helsinki. The study was approved by the Ethical Committee for Research from the Center of Medical Education and Clinical Research (CEMIC) “Norberto Quirno” (Buenos Aires, Argentina). We report all measures and manipulations. No individual subject data were excluded, as data were only recorded if all necessary questions in forms were completed.

### Experimental Situations Design

#### Basic Conflicts Design

The combination of the three claims chosen for our study produces three possible scenarios of conflict. The order in which the claims can appear in each situation cannot be arbitrary: “discovery” involves first possession, while “occupation” can only be performed by a second agent. Then, the three possible conflicts are the following, which will be referred to as the *fundamental* conflicts: (1) a discoverer of a raw material who is challenged by a creator of a new item from said materials, (2) a discoverer of a living territory who is challenged by second person who occupies it, and (3) a creator of a living space who is challenged by another person who occupies it. These three situations depict not only different ownership claims, but also changes in the identity of what is disputed: a manufactured item, a territory, and a habitable construction. A basic set of situations was designed to present the aforementioned conflicts. Characters were always defined as *X* and *Z*, and in that order, for every story:

Discovery versus creation (D vs. C): “*X discovers an amount of a certain material. Z uses the material to manufacture something.*”

Discovery versus occupation (D vs. O): “*X discovers a livable place (e.g., an island, a cave). Z occupies it actively for some time*.”

Creation versus occupation (C vs. O): “*Using freely available materials, X builds a livable space. Z occupies it actively for some time*.”

From now on, we will refer to this set of situations as the *basic situations*. The statements were conceived in the most concise manner still able to unambiguously depict the characters’ claims. For example, the occupier is said to occupy the place or the space “*actively for some time*,” which distinguishes a circumstantial occupation from a case of true adverse possession as understood by common sense (and law). In the C vs. O conflict, the creator is said to build the space from “*freely available materials*” to prevent participant from potentially believing that the raw materials had a previous owner (as in the D vs. C situation).

#### Variations from the Basic Situations

After the set of basic conflicts was determined, variations were designed to tackle our study goal, which was to determine whether resolution by participants depends on the consideration of social validation of property claims. Accordingly, the variations covered two possible details in the conflicts: (1) that the second character was aware of the first character’s claim and (2) that third parties had knowledge of the first character’s claim.

##### Second character’s knowledge

Binary variations of the basic situations were designed that stated whether the second character knew about the first possessor’s claim or not. This element will be referred to as the “*knowledge*” factor, with binary variations having either negative or positive “sign.” As an example, we transcribe here the variations for the D vs. C conflict that describe whether the creator had previous knowledge about the first character’s discovery:

No knowledge by the second character: “*X discovers an amount of a certain material. Z, without knowing about X’s discovery, uses the material to manufacture something*.”

Knowledge by the second character: “*X discovers an amount of a certain material. Z, knowing about X’s discovery, uses the material to manufacture something*.”

##### Third parties’ knowledge

Other persons may acquire knowledge about the actions of the first character in two ways: either by being informed by her or by witnessing her actions. Hence, both possibilities were evaluated in our study and will be referred to as the “*communication*” factor and the “*witnesses*” factor, respectively, with their binary variations having positive or negative signs. Because detailing whether third-parties knew about the claim of the first possessor can drive participants to wonder whether the second character shared such knowledge, it was made clear in the statements that this was not the case. In this sense, variations for these two factors are considered to be based on the “*no knowledge by the second character*” case. The following examples illustrate the variations corresponding to the D vs. C conflict:

No communication to third parties: “*X discovers an amount of a certain material. He does not communicate it to other people. Z, without knowing about X’s discovery, uses the material to manufacture something*.”

Communication from the first character to third parties: “*X discovers an amount of a certain material, and he communicates it to many people. Z, without knowing about X’s discovery, uses the material to manufacture something*.”

No witnesses for the first character’s actions: “*X discovers an amount of a certain material. There are no witnesses of his discovery. Z, without knowing about X’s discovery, uses the material to manufacture something*.”

Presence of witnesses for the first character’s actions: “*X discovers an amount of a certain material, and many people witness his discovery. Z, without knowing about X’s discovery, uses the material to manufacture something*.”

#### Summary of All Situations Tested in the Study

The study involved a total of 21 situations, summarized below:

- Three *basic situations*, one for each of the fundamental conflicts.
- Six variations for the *knowledge* factor, with a pair of negative/positive variations of the factor for each of the fundamental conflicts.
- Six variations for the *communication* factor, with a pair of negative/positive variations of the factor for each of the fundamental conflicts.
- Six variations for the *witnesses* factor, with a pair of negative/positive variations of the factor for each of the fundamental conflicts.

**Table 1** shows the total of volunteers who judged over each situation in the study. The complete set of 21 statements in the original form in Spanish and the corresponding translated versions can be found in the Supplementary Material.

**Table 1:** Summary of participants’ judgments for every situation in the study.

		“Knowledge” factor		“Communication” factor		“Witnesses” factor	
Conflict	Basic situations	(-)	(+)	(-)	(+)	(-)	(+)
D vs. C	**55** 7/48	**54** 9/91	**53** 28/72^∗^	**45** 0/100	**51** 8/92	**45** 2/98	**56** 25/75^∗∗^


D vs. O	**55** 25/30	**59** 24/76	**58** 69/31^∗∗∗^	**45** 20/80	**51** 29/71	**45** 18/82	**46** 46/54^∗^


C vs. O	**55** 47/8	**48** 87/13	**52** 100/0^∗^	**45** 82/18	**56** 89/11	**45** 89/11	**58** 88/12

*The total answers to each of the situations tested in the study are presented in bold. The pair of values below each of these totals represent the percentages of judgments favoring the first character/the second character for each situation. Asterisks indicate pairs of variations for a determined factor and conflict that showed significant differences after two-tailed Chi-square tests (full statistical details presented in the Supplementary Material). The signs “-” and “+” indicate the “sign” of each of the variations. Supplementary Table 1 presents the actual number of judgments favoring each character for each of the situations in the study. Bonferroni corrected *p*-values: ^∗^*p* < 0.016, ^∗∗^*p* < 0.0033, ^∗∗∗^*p* < 0.00033*.

### Questionnaires

After completing the informed consent and providing age and gender data, each participant was faced with the following instructions, after which they were presented with the first case in a separate page:

*Resolution of conflicts: Through the following pages, we will present you with different situations involving two characters, X and Z. Read each of the situations attentively. Some of them might seem similar to the others, but they may present subtle differences. We ask you to consider each of the presented situations as isolated and independent from the previous ones. Answer to the questions you are presented with according to your judgment*.

Case 1. Analyze the following situation:

‘*X discovers an amount of a certain material. Z uses the material to manufacture something*.’

Whose manufactured object (space, or place, depending on the conflict) is it?

*Answer exclusively according to your judgment. You have to decide in favor of one of the two characters, so take your time*.

The participant was allowed to choose either *X* or *Z*. After deciding, they were presented with the following case. Three situations were presented to each volunteer. No answers were recorded unless all three activities were completed and submitted.

We divided the volunteers into two experiments. Experiment I was designed to probe the intuitions of our population regarding the fundamental conflicts that we conceived. Volunteers (*n* = 55, mean age ± SD = 35.3 ± 12.8, females = 61%) received each of the three basic situations in a random order.

In Experiment II, volunteers (*n* = 304; mean age ± SD = 32.9 ± 11.7, females = 60%) judged over a set of three variations from the basic situations. Each participant judged over three cases, one corresponding to each of the fundamental conflicts, and each one of these displaying a variation on a different factor (“*knowledge*,” “*communication*,” or “*witnesses*”), in a random order and combination. For example, a given participant would get a combination like the following: (1) D vs. C: “*no knowledge by the second character*”; (2) C vs. O: “*communication from the first character to third parties*”; and (3) D vs. O: “*presence of witnesses for the first character’s actions*.”

The reason for dividing the study into two experiments, one focusing on the basic statements and the other on the variations, was that if a participant received one of the variations of the studied factors of a conflict, and considered such information for her judgment, such consideration might be “carried over” to a following basic situation devoid of details on such factor. The difference in the number of participants between the two experimental populations derives from the total of statements tested in each experiment: every participant in the first experiment received every basic situation, while a participant in the second experiment received only 3 out of the 18 different situations in the set (three fundamental conflicts, times three factors, times two variations for each factor). This design allowed us to have 51 ± 7 answers/judgments for each situation tested.

### Data Analysis

Analysis of the distribution of binary judgments in each group was performed as follows. First, according to the experiment, a general linear model (GLM) or a general linear mixed model (GLMM) was fitted to the responses data, including all potential factors influencing judgments: age, gender, order of presentation of the stories through the questionnaire, the fundamental conflict involved, and the type and sign of variation (these two only for Experiment II). Then, *factor-null* models were fitted in which each of these factors was individually removed from the original model. Finally, each of these null models was compared to the original model through likelihood ratio tests (LRT). The results of the LRT are reported as the effects of each individual factor on judgments.

Fit of GLM, GLMMs, and LRT was performed in R (R Development Core Team, 2011). Mixed models were performed with the *lme4* package (Bates et al., 2015).

## Results

First, let’s remember our three model situations on which participants judged. In the D vs. C story, a discoverer of raw material is challenged by a creator of a new item from said materials. In the D vs. O story, a discoverer of a living territory is challenged by second character who occupies it. Finally, in the C vs. O story, a creator of a living space is challenged by another character who occupies it.

### Intuitions on the Basic Situations

In Experiment I, we tested the judgments of participants to the three fundamental conflicts. The objective was to define our population basic intuitions regarding the situations that we would further work on Experiment II.

Judgments of the three main dilemmas in Experiment I showed that creators received significantly more favorable decisions (meaning they were more likely to be judged as the owners of the disputed property) than discoverers (87% in favor of the creator; binomial test: *p* < 0.001, RR = 1.745) and occupiers (85% in favor of the creator; *p* < 0.001, RR = 1.709). By contrast, the D vs. O conflict did not show a significant preference in favor of either of the two agents (45% vs. 55% for the discoverer and the occupier, respectively; **Table 1** and Supplementary Table 2).

We fitted all participants’ answers to a GLMM including age, gender, order of presentation of the stories, and finally, the identity of the specific conflict evaluated. The GLMM fit followed by LRT comparing “null models” for each factor revealed that both the fundamental conflict involved (*X*^2^(3) = 69.084, *p* < 0.001) and age (*X*^2^(1) = 6.961, *p* = 0.008) were significant determinants of judgments. On the other hand, neither gender (*X*^2^(1) = 2.268, *p* = 0.132) nor of order of presentation (*X*^2^(1) = 0.215, *p* = 0.642) had any effects on the outcome of decisions.

The three fundamental conflicts, then, represent very different scenarios on which to test the hypothesis of Experiment II: one clearly determined in favor of the first agent in the sequence (the creator over the occupier), one in favor of the second agent (again the creator, over the discoverer), and one with no population consensus on its resolution (the discoverer vs. the occupier).

### Second Agent’s Knowledge of the First Agent’s Claim

The first question in Experiment II was whether the fact that the second agent in the stories knew or not about the first agent’s claim for ownership was a determinant of volunteer’s resolution of the conflicts.

**FIGURE 1:**
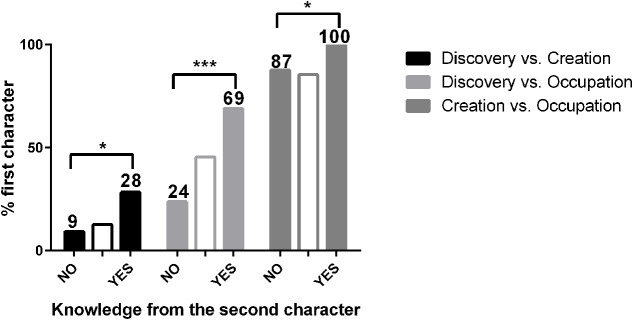
Proportion of judgments favoring the first character when considering the “*knowledge*” factor: whether the second character was aware of the first possessor’s claim or not. The intermediate blank bars are included as means of illustration of the judgments in the basic situations on which each variation is based. GLM-LRT analysis revealed a significant effect of the “*knowledge*” factor (*X*^2^(3) = 41.49, *p* < 0.001). Asterisks indicate significant differences between variations from Chi-square tests (Supplementary Table 3). Bonferroni corrected *p*-values: ^∗^*p* < 0.016, ^∗∗∗^*p* < 0.00033.

The results showed that information about the knowledge of the second character concerning the first character’s actions had a strong effect on judgments (**Figure 1**). Judgments favoring the first character increased nearly threefold in the D vs. C (from 9% to 28% in favor of the discoverer) and the D vs. O cases (from 24% to 69% in favor of the discoverer), and over 10% in the C vs. O case (from 87% to 100% in favor of the creator), for the statements that reported that the second character knew of the first character’s claim in comparison to the ones stating that the second character was not aware of it (**Table 1**).

We fitted a GLM to the volunteers’ answers including age, gender, order of presentation of the stories, the particular fundamental conflict involved, and whether the second character in the stories knew about the first’s claim. We added an interaction term to the model considering that the “*knowledge*” factor could have different effects according to the fundamental conflict considered. The GLM-LRT analysis confirmed a significant effect of the “*knowledge*” factor (*X*^2^(3) = 41.49, *p* < 0.001) and no interaction with the fundamental conflicts (*X*^2^(2) = 2.910, *p* = 0.233). Neither age (*X*^2^(1) = 0.034, *p* = 0.855), nor gender (*X*^2^(1) = 0.012, *p* = 0.913), nor order of presentation (*X*^2^(1) = 1.429, *p* = 0.232) had significant effects on decisions.

In short, these results show that in everyday intuitions people judge that if a person acts on stuff and claims property of it based on her actions, her rights to own the stuff are diminished when she was aware that there was a previous agent claiming ownership over it. Instead, if she was ignorant of the first agent’s actions, her claim over the stuff holds a stronger value.

### Third-Parties’ Knowledge of the First Agent’s Actions

The second question Experiment II wanted to answer was whether knowledge from third parties about the claim of a first agent would modify volunteers’ decisions regarding ownership challenges from a second agent.

**FIGURE 2:**
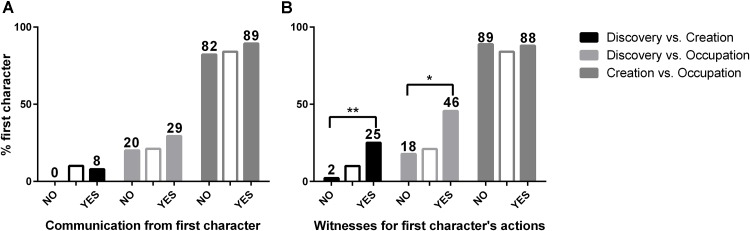
Proportion of judgments favoring the first character when considering **(A)** the “*communication*” factor: whether the first character communicated her actions to third-parties or not; and **(B)** the “*witnesses*” factor: whether third-parties witnessed the first character’s actions or not. The intermediate blank bars are included as means of illustration of the judgments in the “*no knowledge by the second character*” situation on which each variation is based (see section “Materials and Methods”). GLM-LRT analyses for each factor revealed significant effects of the “*witnesses*” factor (*X*^2^(3) = 22.229, *p* < 0.001), but none of the “*communication*” factor (*X*^2^(3) = 6.890, *p* = 0.075). Asterisks indicate significant differences between variations from Chi-square tests (Supplementary Tables 4, 5). Bonferroni corrected *p*-values: ^∗^*p* < 0.016, ^∗∗^*p* < 0.0033.

Information on whether the first character in the stories had communicated her claim to others or not had no effect on participants judgments (**Figure 2A** and **Table 1**). A GLM fit identical to the described in the above subsection, followed by LRT comparisons, reported no effect of the “*communication*” factor (*X*^2^(3) = 6.890, *p* = 0.075), and no interaction according to fundamental conflicts (*X*^2^(2) = 3.091, *p* = 0.213). We found a significant effect of age (*X*^2^(1) = 6.843, *p* = 0.009), but none of gender (*X*^2^(1) = 1.342, *p* = 0.247) nor of order of presentation of the stories (*X*^2^(1) = 0.223, *p* = 0.637).

The presence or absence of witnesses for the actions of the first character, on the other hand, was a major determinant of judgments (**Figure 2B** and **Table 1**). Situations stating that the first character’s claim was witnessed by many people significantly increased judgments in her favor in the D vs. C (from 2% to 25% in favor of the discoverer) and the D vs. O conflicts (from 18% to 46% in favor of the discoverer). No significative effect was found in the C vs. O conflict, which already showed a high proportion of decisions favoring the creator from the basic situation. A GLM-LRT analysis reported a significant effect of the “*witnesses*” factor (*X*^2^(3) = 22.229, *p* < 0.001), and a significant interaction with the fundamental conflict involved (*X*^2^(2) = 7.464, *p* = 0.024). Neither age (*X*^2^(1) = 1.600, *p* = 0.206), nor gender (*X*^2^(1) = 3.058, *p* = 0.080), nor order of presentation (*X*^2^(1) = 0.014, *p* = 0.906) had significant effects on decisions.

Altogether, these results indicate that validation from third parties can reinforce the first agents’ claims or, inversely, weaken the second agents’ challenges, but only when this validation is achieved by factual confirmation. While merely communicating her claim to third parties did not make the first agent’s position any stronger, counting on witnesses for her actions provided her claims with a better protection from subsequent challenges.

## Discussion

In the present study we show that basic intuitions on the resolution of ownership conflicts can be biased according to social and “institutional” considerations that go beyond the analysis of individual claims. In short, we have found that an agent’s previous knowledge about the history of ownership of a given item weakens his subsequent claim over it, even if her actions present a strong case for ownership. Also, the validation by witnesses of an agent’s claim over something provides defense against a second agent’s dispute of such ownership. Such protection, however, cannot be achieved by the mere communication of property claims to third parties.

Ownership rights can be acquired either *ex novo* or, most commonly, through transference from a previous owner. People recognize many valid ways in which transference of property can be achieved, as well as many others which are unacceptable or illegal (but which may result in transference of ownership anyway). Frequently, ownership rights are contested, thus creating a conflict and requiring a resolution that is not self-evident from the start. When the veracity and legitimacy of the agents making opposing ownership claims is undisputed, an unambiguous solution must be carefully constructed.

Neither actual nor previous possession can be used as the ultimate criteria for deciding ownership in a conflict of parties. One key question then is “who has a better right?” Is it the case that we all observe an ideal hierarchy of principles that inform us on how to adjudicate ownership? The resolution of such conflicts has been studied from different angles in the literature. Kanngiesser et al. (2010) showed that both adults and children approved ownership transfers of clay figures modeled by one person, after another person had worked on them so as to create a new shape, thus indicating that creation may trump first possession (of the clay). Friedman (2010) proved that people value the intention to possess something (viewed as the effort invested in such task) over physical possession *per se* (Palamar et al., 2012). Rochat et al. (2014) studied the answers of children to ownership dilemmas, and found that they favor first possession in most cases. Shotland and Hyers (2000) have shown that Common Law regarding adverse possession seems to be based on current social considerations, while DeScioli and Karpoff (2015), on the other hand, have found that people often do not consider property conflicts in line with the law’s reasoning. In brief, there are evidences that ownership rights are judged according to principles that include both first possession and the merit of the parties involved. Our first experimental group who judged over the basic situations, for example, reflected the prevalence of “creation” as a strong claim for ownership.

But property is also a social and institutional act, in the sense of Searle (1995). As we hinted in the section “Introduction,” ownership is a construct which demands not only relevant actions to acquire such property, but also to be backed and validated by agents from the social environment. The objective of this study was to determine if this also holds true for participants’ judgments to ownership conflicts.

In the first place, we found that people consider transgression of property claims less offensive when the transgressor is unaware of the actions of the original claimant/owner. The second claimant’s knowledge of the original possessor was a major determinant of judgments in the all situations tested. A simple explanation may be that people see the informed second character as someone who willfully and unrightfully seeks to take property from the first character. People seem to consider that it is not acceptable to *intentionally* and *consciously* transgress others’ ownership rights. For example, Millar et al. (2014) have shown that adults would accept to sacrifice their own property, but not another person’s, in order to save a third’s more valuable stuff, considering the respect of thirds’ property over utilitarian implications. This is consistent with real-world situations in the fact that, in some jurisdictions, authorities have rejected claims to adverse possession when they originated from intentional trespass (Austin, 2014). It is interesting nevertheless that 65% of participants in our experiment considered that it was OK for the creator character to take ownership of the raw materials from the discoverer (and own the created object), even when she knew that someone held a claim over it. This reinforces the idea that some claims are not considered as strong as others for establishing property rights.

Establishing ownership also requires that certain social norms are met to allow validation of property rights from other agents, which in turn prevents both willful and unintentional transgressions of such rights, and hence maintains social peace and reduces unnecessary conflicts (Rose, 1985). The best method to achieve this is to make ownership known to others by appropriate means, and in this sense publicizing one’s property claim may be seen as attempting to make explicit the intention to own and control (Fuller, 1969).

Officialization of established property is a basic legal requirement in every society ruled by law (Rose, 1985). This is only logical. In the words of Austin, if what the concept of “ownership involves is the idea that third parties have obligations in relation to the owner … then publicity would demand that these third parties know that something is owned rather than unowned” (Austin, 2014, p. 93). Publicity, therefore, is essential for ownership claims being respected by others. Our study demonstrated that counting on witnesses for the actions of the first character reinforces her claims, but that simple communication of such acts is not as relevant. These results suggest that ownership claims demand a specific level of acknowledgment from the public to be validated and hence receive support against potential future claimants. We cannot discard the possibility that these and other forms of social validation interact to some degree with the previously discussed “knowledge” factor: participants may assess the assertion that the second character is unaware of the original agent’s claim as being more or less likely to be true according to the specific method of validation. Although we cannot control such assumptions from participants, the statement in the “communication” and “witnesses” variations that the second character did not know about the history of the disputed object leads us to assume that such an interaction would, in average, be filtered out.

The present work opens additional questions toward a deeper understanding of human intuitions on property. An interesting subject to address regards the ethical considerations that are involved in the establishment of property (for a comprehensive summary of the philosophical analysis of ownership, see Waldron, 2016). In his *First Treatise on Government*, Locke (1988) proposed that individuals gain private ownership by mixing his labor with nature. On the other hand, whenever someone makes something new available to society, or idle resources are put to work, a utilitarian consideration may also demand property rights. Finally, social or individual needs may lead to the assignment of property for the sake of increasing fairness (Rawls, 1999). In essence, these considerations are intrinsic to the concept of property as it is understood by both Common and International Law. The three traditional claims for ownership that are used in the study differ significantly in such moral meanings. Discovery implies that new, presumably valuable stuff is made available, and therefore it increases the total net value of society in utilitarian terms. Occupation may be seen as reflecting the individual’s need or a social necessity to put to work otherwise idle resources, and may thus promote equality. Creation demands investment of physical and creative labor, and it represents individualistic, Lockean values at their best. These differences added another layer of complexity to our set of conflicts, thus increasing the range of situations on which to test the factors in our study. However, this also means that it is impossible to disentangle the potential weights of each of such ethical considerations in our volunteers’ judgments – but, as stated above, these are nevertheless fundamental to the nature of ownership. Further studies should be carried out to measure the relative weights of such considerations in the settling of property in similar conflicts between parties.

We believe this study provides novel elements to consider in the study of ownership notions and reasoning. To our knowledge, this is the first work studying these features in the resolution of property dilemmas. Further studies should determine whether these normative considerations are already present in children, which have been shown to resolve conflicts following strategies that are not always identical to those of adults. Further work in this area of common sense notions about property, on how we come to own things and interchange them, and on how ownership rights can be obtained and contested will help us understand our social behavior and the evolution of ever changing social norms and law. In words of Friedrich Hayek:

*While property is initially a product of custom, and jurisdiction and legislation have merely developed it in the course of millennia, there is then no reason to suppose that the particular forms it has assumed in the contemporary world are final. Traditional concepts of property rights have in recent times been recognized as a modifiable and very complex bundle whose most effective combinations have not yet been discovered in all areas* (Hayek, 1988, p. 36).

## Author Contributions

LC designed and carried out the study, analyzed the results, discussed the findings, and wrote the article. GF participated in the design and discussion of the study and wrote the article. AC participated in the design and discussion of the study. MS supervised and designed the study, discussed the findings, and revised the article.

## Conflict of Interest Statement

The authors declare that the research was conducted in the absence of any commercial or financial relationships that could be construed as a potential conflict of interest.
