# Supplementary material for: Social Validation Influences Individuals’ Judgments about Ownership
Source: Front Integr Neurosci. 2018 Jan 30;12:2. doi: 10.3389/fnint.2018.00002 (PMC5797628; doi:10.3389/fnint.2018.00002)
Supplement: Supplementary file 1 [file Data_Sheet_1.PDF]

Supplementary Material

Sets of Original Statements in Spanish and Corresponding English Translations

Below, all the statements and variations are presented in Spanish, followed with the translated version (in italics). For the sake of simplification of the text, we have used the following abbreviations: the *"knowledge"* factor is referred as Kf, the *"communication"* factor as Cf, and the *"witnesses"* factor as Wf. Each factor's variations are indicated as having either negative or positive sign.

Discovery versus Creation.

Basic situation: "X descubre una cantidad de un determinado material. Z lo usa para fabricar algo."

"X discovers an amount of a certain material. Z uses the material to fabricate something."

Kf (-): "X descubre una cantidad de un determinado material. Z, sin saber que el lugar fue descubierto por X, lo usa para fabricar algo."

"X discovers an amount of a certain material. Z, without knowing about X's discovery, uses the material to fabricate something."

Kf (+): "X descubre una cantidad de un determinado material. Z , sabiendo que el material fue descubierto por X, lo usa para fabricar algo."

"X discovers an amount of a certain material. Z, knowing about X's discovery, uses the material to fabricate something."

Wf (-): "X descubre una cantidad de un determinado material. No hay testigos de su descubrimiento. Z, sin saber que el lugar fue descubierto por X, lo usa para fabricar algo."

"X discovers an amount of a certain material. There are no witnesses of his discovery. Z, without knowing about X's discovery, uses the material to fabricate something."

Wf (+): "X descubre una cantidad de un determinado material, y muchas personas son testigos de su descubrimiento. Z, sin saber que el lugar fue descubierto por X, lo usa para fabricar algo."

"X discovers an amount of a certain material, and many people witness his discovery. Z, without knowing about X's discovery, uses the material to fabricate something."

Cf (-): "X descubre una cantidad de un determinado material. No se lo comunica a otras personas. Z, sin saber que el lugar fue descubierto por X, lo usa para fabricar algo."

"X discovers an amount of a certain material. He does not communicate it to other people. Z, without knowing about X's discovery, uses the material to fabricate something."

Cf (+): "X descubre una cantidad de un determinado material, y se lo comunica a muchas personas. Z, sin saber que el lugar fue descubierto por X, lo usa para fabricar algo."

"X discovers an amount of a certain material, and he communicates it to many people. Z, without knowing about X's discovery, uses the material to fabricate something."

Discovery versus Occupation.

Basic situation: "X descubre un lugar habitable (por ejemplo una cueva, o una isla). Z lo ocupa activamente durante un tiempo."

"X discovers a habitable place (e.g. an island, a cave). Z occupies it actively for some time."

Kf (-): "X descubre un lugar habitable (por ejemplo una cueva, o una isla). Z, sin saber que el lugar fue descubierto por X, lo ocupa activamente durante un tiempo."

"X discovers a habitable place (e.g. an island, a cave). Z, without knowing about X's discovery, occupies it actively for some time."

Kf (+): "X descubre un lugar habitable (por ejemplo una cueva, o una isla). Z, sabiendo que el lugar fue descubierto por X, lo ocupa activamente durante un tiempo."

"X discovers a habitable place (e.g. an island, a cave). Z, knowing about X's discovery, occupies it actively for some time."

Wf (-): "X descubre un lugar habitable (por ejemplo una cueva, o una isla). No hay testigos de su descubrimiento. Z, sin saber que el lugar fue descubierto por X, lo ocupa activamente durante un tiempo."

"X discovers a habitable place (e.g. an island, a cave). There are no witnesses of his discovery. Z, without knowing about X's discovery, occupies it actively for some time."

Wf (+): "X descubre un lugar habitable (por ejemplo una cueva, o una isla), y muchas personas son testigos de su descubrimiento. Z, sin saber que el lugar fue descubierto por X, lo ocupa activamente durante un tiempo"

"X discovers a habitable place (e.g. an island, a cave), and many people witness his discovery. Z, without knowing about X's discovery, occupies it actively for some time."

Cf (-): "X descubre un lugar habitable (por ejemplo una cueva, o una isla). No se lo comunica a otras personas. Z, sin saber que el lugar fue descubierto por X, lo ocupa activamente durante un tiempo."

"X discovers a habitable place (e.g. an island, a cave). He does not communicate it to other people. Z, without knowing about X's discovery, occupies it actively for some time."

Cf (+): "X descubre un lugar habitable (por ejemplo una cueva, o una isla), y se lo comunica a muchas personas. Z, sin saber que el lugar fue descubierto por X, lo ocupa activamente durante un tiempo."

"X discovers a habitable place (e.g. an island, a cave), and he communicates it to many people. Z, without knowing about X's discovery, occupies it actively for some time."

Creation versus Occupation.

Basic situation: "Con materiales de libre disponibilidad, X construye un espacio habitable. Z lo ocupa activamente durante un tiempo."

"Using freely available materials, X builds an habitable space. Z occupies it actively for some time."

Kf (-): "Con materiales de libre disponibilidad, X construye un espacio habitable. Z, sin saber que el espacio fue construido por X, lo ocupa activamente durante un tiempo."

"Using freely available materials, X builds an habitable space. Z, without knowing that X built the space, occupies it actively for some time."

Kf (+): "Con materiales de libre disponibilidad, X construye un espacio habitable. Z, sabiendo que el espacio fue construido por X, lo ocupa activamente durante un tiempo."

"Using freely available materials, X builds an habitable space. Z, knowing that X built the space, occupies it actively for some time."

Wf (-): "Con materiales de libre disponibilidad, X construye un espacio habitable. No hay testigos de su construcción. Z, sin saber que el espacio fue construido por X, lo ocupa activamente durante un tiempo."

"Using freely available materials, X builds an habitable space. There are no witnesses of his creation. Z, without knowing that X built the space, occupies it actively for some time."

Wf (+): "Con materiales de libre disponibilidad, X construye un espacio habitable, y muchas personas son testigos de su construcción. Z, sin saber que el espacio fue construido por X, lo ocupa activamente durante un tiempo."

"Using freely available materials, X builds an habitable space, and many people witness his creation. Z, without knowing that X built the space, occupies it actively for some time."

Cf (-): "Con materiales de libre disponibilidad, X construye un espacio habitable. No se lo comunica a otras personas. Z, sin saber que el espacio fue construido por X, lo ocupa activamente durante un tiempo."

"Using freely available materials, X builds an habitable space. He does not communicate it to other people. Z, without knowing that X built the space, occupies it actively for some time."

Cf (+): "Con materiales de libre disponibilidad, X construye un espacio habitable, y se lo comunica a muchas personas. Z, sin saber que el espacio fue construido por X, lo ocupa activamente durante un tiempo."

"Using freely available materials, X builds an habitable space, and he communicates it to many people. Z, without knowing that X built the space, occupies it actively for some time."

Example questionnaire in Spanish and English translation

After completing the informed consent, and filling age and gender data, participants were presented with the following instructions:

Spanish original:

"Resolución de un conflictos

A lo largo de las siguientes páginas, te vamos a ir presentando diferentes situaciones protagonizadas por dos personajes, X y Z.

Leé cada una de las situaciones con atención. Algunas pueden parecerse entre sí, pero pueden presentar diferencias sutiles.

Te pedimos que consideres cada una de las situaciones que te presentemos como aislada e independiente de las anteriores.

Contestá a las preguntas que vayan apareciendo de acuerdo a tu criterio."

English translation:

"Resolution of conflicts

Through the following pages, we will present you with different situations involving two characters, X and Z.

Read each of the situations attentively. Some of them might seem similar to the others, but they may present subtle differences.

We ask you to consider each of the presented situations as isolated and independent from the previous ones.

Answer to the questions you are presented with according to your judgment."

The next page presented the volunteers with the first situation, and asked them to judge over the conflict:

Spanish original:

"Caso 1

Analizá la siguiente situación:

X descubre una cantidad de un determinado material.

Z lo usa para fabricar algo.

¿De quién es el objeto fabricado?

Respondé únicamente de acuerdo a tu criterio. Tenés que decidir por uno de los dos, así que tomate tu tiempo.

☐ *De X*

☐ *De Z "*

English translation:

"Case 1.

Analyze the following situation:

X discovers an amount of a certain material.

Z uses the material to manufacture something.

Whose manufactured object is it?

Answer exclusively according to your judgment. You have to decide in favor of one of the two characters, so take your time.

☐ *X's*

☐ *Z's"*

After responding to the question, the participants received the following case in the same format.

Supplementary Tables

Supplementary Table 1

	Basic situations	*“Knowledge” factor*		*“Communication” factor*		*“Witnesses” factor*	
Conflict	*N/A*	*(-)*	*(+)*	*(-)*	*(+)*	*(-)*	*(+)*
D vs. C	**55**<br>7/48	**54**<br>5/49	**53**<br>15/38*	**45**<br>0/45	**51**<br>4/47	**45**<br>1/44	**56**<br>14/42**
D vs. O	**55**<br>25/30	**59**<br>14/45	**58**<br>40/18***	**45**<br>9/36	**51**<br>15/36	**45**<br>8/37	**46**<br>21/25*
C vs. O	**55**<br>47/8	**48**<br>42/6	**52**<br>52/0*	**45**<br>37/8	**56**<br>50/6	**45**<br>40/5	**58**<br>51/7

Summary of Participants Judgments for Every Situation in the Study. The table shows the total of answers to each of the situations tested in the study, in bold. The pair of numbers below each total represent the number of judgments favoring the first character / number of judgments favoring the second character for each situation. Asterisks indicate pairs of variations for a determined factor and conflict that showed significant differences after two-tailed Chi Square tests (full statistical details presented in the Supplementary Material). The signs “-” and “+” indicate the “sign” of each of the variations.

Bonferroni corrected *p* values: * $p < .016$, ** $p < .0033$, *** $p < .00033$

Supplementary Table 2

Judgments in the Basic Situations

**Conflict**	**1º/T**	**Binomial**
D vs. C	7/55	p < .0001<br>RR 1.7455
D vs. O	25/55	p .295<br>RR 1.0909
C vs. O	47/55	p < .0001<br>RR 1.7091

Note. Judgments favoring the first possessor over the total answers (*1º/T*) are shown for each basic conflict. Statistical analysis of within-conflicts distributions were analyzed through binomial tests.

Supplementary Table 3

Judgments in the "Knowledge" Factor Variations

Conflict	Knowledge		Comparison
	-	+	
D vs. C	5/54	15/53	p = .0115<br>$\chi^2 = 6.382$<br>RR = 0.4439
D vs. O	14/59	40/58	p < .0001<br>$\chi^2 = 24.08$<br>RR = 0.3630
C vs. O	42/48	52/52	p = .0085<br>$\chi^2 = 6.915$<br>RR = 0.4468

Note. Shown are the total of judgments favoring the first possessor over the total answers in the two variations of the *"knowledge"* factor. The last column show the results of the two-tailed Chi Square comparisons between situations within each conflict.

Supplementary Table 4

Judgments in the "Communication" Factor Variations

**Conflict**	**Communication**		**Comparison**
	-	+	
D vs. C	0/45	4/51	p = .0550<br>χ^2 = 3.683<br>RR 1.957
D vs. O	9/45	15/51	p = .2879<br>χ^2 = 1.129<br>RR = 1.250
C vs. O	37/45	50/56	p = .3072<br>χ^2 = 1.043<br>RR = 1.341

Note. Shown are the total of judgments favoring the first possessor over the total answers in the two variations of the *"communication"* factor. The last column show the results of the two-tailed Chi Square comparisons between situations within each conflict.

Supplementary Table 5

Judgments in the "Witnesses" Factor Variations

**Conflict**	**Witnesses**		**Comparison**
	-	+	
D vs. C	1/45	14/56	p = .001<br>χ^2 10.24<br>RR = 1.911
D vs. O	8/45	21/46	p = .004<br>χ^2 = 8.140<br>RR = 1.796
C vs. O	40/45	51/58	p = .880<br>χ^2 = 0.022<br>RR = 0.961

Note. Shown are the total of judgments favoring the first possessor over the total answers in the two variations of the *"witnesses"* factor. The last column show the results of the two-tailed Chi Square comparisons between situations within each conflict.
